# Acidocétose diabétique chez l'adulte à l'Hôpital Sendwe de Lubumbashi: à propos de 51 cas

**DOI:** 10.11604/pamj.2014.17.324.3545

**Published:** 2014-05-01

**Authors:** Placide Kambola Kakoma, David Mulumba Kadiebwe, Axel Mbuyu Kayembe, Prosper Kashindi Makonga, Marcellin Bugeme, Olivier Mukuku

**Affiliations:** 1 Faculté de Médecine, Université de Lubumbashi, République Démocratique du Congo

**Keywords:** Acidocétose diabétique, hyperglycémie, cétonurie, Lubumbashi, diabetic ketoacidosis, hyperglycemia, ketonuria, Lubumbashi

## Abstract

L'acidocétose diabétique (ACD) est une décompensation métabolique sévère du diabète sucré. L'objectif de notre étude est de Décrire les aspects épidémiologiques, cliniques et évolutifs de l'acidocétose diabétique chez l'adulte à Lubumbashi (République Démocratique du Congo). Il s'agit d'une étude prospective descriptive réalisée du 1er janvier 2011 au 31 décembre 2012 incluant tous les patients âgés de 18 ans ou plus admis pour acidocétose diabétique dans le service de Médecine Interne de l'hôpital Jason Sendwe de Lubumbashi (République Démocratique du Congo). L’échantillon était exhaustif fait de 51 patients chez qui nous les paramètres épidémiologiques, cliniques et évolutifs étaient étudiés. La prévalence de l'acidocétose diabétique au cours de l’étude a été de 5% et elle a constitué la plus fréquente des complications métaboliques aigues du diabète avec 37,2% des cas. L’âge moyen des patients était de 44,8 ans allant de 20 à 79 ans et les hommes ont été plus touchés (58,8%) avec un sexe ratio de 1,42. L'examen clinique a révélé la respiration de Kussmaul dans 80,4%, des signes de déshydratation dans 76,5% et une odeur cétonique de l'haleine dans 56,9% et 58,8% de nos patients étaient admis dans un tableau de coma. L'infection était en tête des causes de la décompensation acidocétosique (54,9%) suivi de la mauvaise observance thérapeutique (29,4%). La durée moyenne du séjour hospitalier dans notre série a été de 9,3 jours et l'affection a été grevée d'un taux de mortalité de 27,5%. Cette complication métabolique aigue du diabète reste donc relativement fréquente et redoutable dans notre milieu avec une prévalence de 5% et un taux de mortalité de 27,5%. Une conscientisation des patients diabétiques et une amélioration de la prise en charge s'avèrent donc urgente.

## Introduction

En République démocratique du Congo (RDC), le diabète est une réalité évolutive. Jadis réputée rare, elle est aujourd'hui très prévalente et touche toutes les couches de la population y compris les plus défavorisés. L'absence de politique de dépistage dans notre pays en font un véritable défi de santé publique au regard des complications liées à son évolution naturelle ou à une prise en charge inadéquate. En effet, si le diagnostic de la maladie est assez aisé, l'absence de politique de dépistage en RDC fait découvrir la maladie le plus souvent tardivement, devant des complications inévitables qui tournent souvent à la tragédie. Au nombre de ces complications citons l'acidocétose diabétique (ACD) qui est définie comme une décompensation métabolique sévère du diabète sucré, caractérisée par une hyperglycémie pouvant varier de 11 mmol/l à des valeurs extrêmes, un pH artériel inférieur à 7,3, un taux de bicarbonates plasmatiques inférieur à 15 mmol/l et une cétonémie ou cétonurie. L'acidocétose est caractérisée par une acidose métabolique à trou anionique augmenté [1, 2].

Son incidence dans le monde est estimée entre 4,6 et 8 épisodes pour 1000 patients diabétiques et elle représente environ 4 à 9% des causes d'hospitalisation des diabétiques [2]. Dans notre pays, une étude menée en 2002 à l'Hôpital Général de Référence de Kinshasa rapportait une prévalence de 29,2% [3]. Le taux de mortalité dont elle est grevée est en moyenne inférieur à 5% avec des extrêmes allant de 0 à plus de 15% [2].

## Méthodes

L’étude s'est déroulée au niveau du service de médecine interne de l'hôpital Jason Sendwe de Lubumbashi (République Démocratique du Congo). Il s'agit d'une étude prospective descriptive menée du 1er janvier 2011 au 31 décembre 2012. Selon le « Joint British Diabetes Societies », le diagnostic d'acidocétose repose sur les critères biochimiques suivants [4]: une hyperglycémie supérieure à 11 mmol/l ou un diabète sucré connu, un pH veineux strictement inférieur à 7,30 et/ou taux de bicarbonates sanguins (HCO3-) strictement inférieur à 15 mmol/l et une cétonémie ≥3 mmol/l ou une cétonurie >2 croix à la bandelette urine. Les dosages plasmatiques du pH, des bicarbonates et des cétones n’étant pas systématiquement réalisés dans notre milieu, nous avons retenu comme critères diagnostiques d'inclusion: une hyperglycémie >11 mmol/l ainsi que la présence d'une cétonurie associée à une glycosurie. Ainsi, nous avons inclus dans cette étude tous les patients âgés d'au moins 18 ans hospitalisés au service de Médecine interne et qui répondaient aux critères diagnostiques ci-haut cités.

Les données étaient recueillies à l'aide d'un questionnaire sur base des dossiers de patients hospitalisés au service de Médecine Interne de l'hôpital Jason Sendwe pour acidocétose diabétique durant la période de notre étude. Les paramètres étudiés étaient: le sexe, l’âge, le motif de consultation, les signes à l'examen clinique, les causes de décompensation, la durée d'hospitalisation et l'issue. La saisie et l'analyse de données ont été effectuées à l'aide du logiciel Epi info 2011 (version 7.0.8.3).

## Résultats

**Figure 1:**
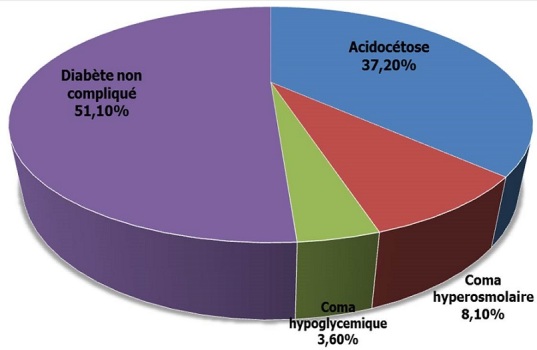
Place de l'acidocétose dans les complications du diabète sucré

Sur un total de 1020 patients hospitalisés dans le service de Médecine interne durant la période d’étude, 51 l'ont été pour acidocétose diabétique, soit une prévalence hospitalière de 5%. Au cours de cette période, 137 patients ont été admis pour diabète sucré et 67 d'entre eux soit 48,9% ont présentés des complications métaboliques aigues dont l'acidocétose diabétique dans 76,1% (51/67) comme le montre la Figure 1.

L’âge moyen de nos patients était de 44,8 ans avec des extrêmes de 20 et 79 ans, près de la moitié (49%) de nos patients avait moins de 40 ans. Le sexe masculin était le plus prédominant (58,8%) avec un sex-ratio de 1,42 (Tableau 1). Les manifestations cliniques les plus souvent notées étaient représentées par la respiration de Kussmaul (80,4%), la déshydratation (76,5%), le coma (58,8%) et l'haleine cétonique (56,9%). Les troubles digestifs, l'hypotension artérielle et la polyuro-polydipsie étaient présents respectivement dans 25,5%, 13,7% et 11,8% des cas comme le montre le Tableau 1.

**Tableau 1:** Répartition des patients suivant les aspects épidémio-cliniques et évolutifs

Paramètre		Effectif (n = 51)	Pourcentage
**Age**			
	≤29 ans	9	17,6
	30–39 ans	16	31,4
	40–49 ans	7	13,7
	50–59 ans	9	17,6
	60–69 ans	6	11,8
	≥ 70 ans	4	7,8
**Sexe**			
	Masculin	30	58,8
	Féminin	21	41,2
**Signes cliniques**			
	Respiration de Kussmaul	41	80,4
	Déshydratation	39	76,5
	Haleine cétonique	29	56,9
	Coma	30	58,8
	Troubles digestifs	13	25,5
	Hypotension	7	13,7
	Polyurie-polydipsie	6	11,8
	Hypothermie	6	11,8
**Facteurs déclenchants**			
	Infections	28	54,9
	Mauvaise observance thérapeutique	15	29,4
	Ignorance de la maladie	6	11,8
	Non élucidé	2	3,9
**Evolution**			
	Guérison	33	64,7
	Décès	14	27,5
	Sortie contre avis médical	4	7,8

**Figure 2:**
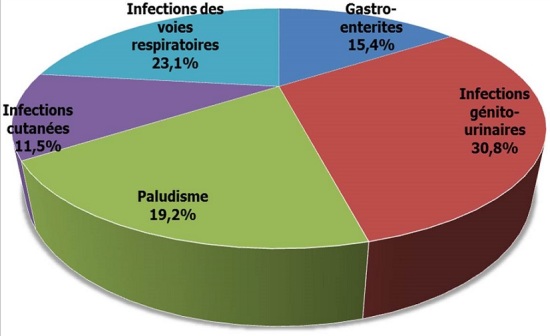
Type d'infection dans la décompensation acidocétosique

Les infections ont été la principale cause de décompensation de la maladie avec 54,9%, suivi de la mauvaise observance thérapeutique (29,4%) et de la méconnaissance de la maladie (11,8%). Chez 2 patients de la série, la cause de décompensation n'a pas été élucidée après toutes les investigations. Les infections génito-urinaires ont représenté 30,8% des causes infectieuses à l'origine de la décompensation de la maladie, suivie de l'infection des voies respiratoires à 23,1%, du paludisme à 19,2%, des gastro-entérites à 15,4%, enfin des infections des parties molles et de la peau à 11,5% (Figure 2). Le séjour hospitalier moyen était de 9,3 jours avec des extrêmes allant de 5 à 20 jours. L’évolution était favorable dans 64,7% de cas, nous avons enregistré 14 décès soit 27,5% et 4 patients ont demandé de quitter l'hôpital malgré un avis contraire du personnel médical.

## Discussion

Dans notre série, la prévalence hospitalière de l'acidocétose diabétique est de 5%. Des chiffres comparables sont rapportés ailleurs sur la scène continentale: 5,9% au Bénin [5], 6,6% au Maroc [6]. Sur l'ensemble de patients diabétiques admis au service, 37,2% ont présenté l'acidocétose diabétique. Cette prévalence est supérieure à celle trouvé au CHU de Yopougon en Abidjan (Côte d'ivoire) par Koffi qui rapporte 26,8% d'acidocétose sur un total de 1 340 diabétiques hospitalisés entre 2002 et 2006 [7].

Les données de la littérature suggèrent que l'acidocétose est la plus fréquente des complications métaboliques aigues du diabète et constitue 4 à 9% des motifs de consultation des patients diabétiques [1]. Notre étude s'aligne dans la droite ligne de ce constat; elle a été la plus fréquente des complications métaboliques aigues du diabète sucré 37,2% (51/137), suivi du coma hyperosmolaire (8,10%) et du coma hypoglycémique (3,6%). Ces résultats sont superposables à ceux Diakite [8] et Diarra [9] qui rapportent respectivement des fréquences d'acidocétose diabétique de 52,5% et 62,2% contre une fréquence respectivement de 37,3%, 29,7% de coma hypoglycémique et de 10,2%, 8,1% de coma hyperosmolaire.

Le sexe masculin était prédominant dans notre étude (58,8%) avec un sex-ratio de 1,42. Cette prédominance du sexe masculin est rapportée par certains auteurs [10–14]; par contre d'autres rapportent une prédominance féminine [9, 15]. Compte tenu de ces divergences, nous ne pouvons pas dire que l'acidocétose diabétique est plus l'apanage des hommes que des femmes. Même les études menées chez l'enfant rapportent des divergences en ce qui concerne la prédominance d'un sexe par rapport à un autre [12, 16–18]. L'influence du sexe peut dépendre de la période de consultation, de la population en présence et de l'attention du diabétique sur lui-même.

L’âge moyen de 44,8 ans. Il est identique à celui rapporté par Monabeka [14] et légèrement supérieur à ceux calculés par Randall et Elmehdawi qui trouvent respectivement 40,8±13,3 ans et 38,3±18,5 ans [11, 12]. Dans la série de Koffi, l’âge moyen était de 49,15 ± 13,49 ans [7].

A l'examen clinique, la respiration de Kussmaul, la déshydratation, l'altération de la conscience et l'odeur acétonémique de l'haleine ont été les principaux signes retrouvés avec respectivement des fréquences de 80,4%, 76,5%, 58,8% et 56,9%. Pour Diarra, la dyspnée de Kussmaul et l'haleine cétonique étaient présentes avec respectivement 100% et 69,5% [9]. Diakite rapporte quant à lui des fréquences respectives de 6,8% et 3,4% pour ces deux signes [8]. Dans notre série, plus d'un patient sur deux (58,8%) était admis dans un tableau de coma. Nous pensons que ce taux élevé de coma serait dû au fait que les malades qui consultent cet hôpital sont généralement référés d'une autre institution hospitalière, habituellement de petits centres de la périphérie de la ville où une prise en charge adéquate n'est pas toujours garantit. Nous pouvons donc fortement supposer que la décompensation acidocétosique qui avait certainement débuté par les signes d'appel de cétose ou d'acidose (douleurs abdominales, douleurs thoraciques, nausées et vomissements, agitation etc.) aura évolué vers l'installation d'un coma, faute de prise en charge adéquate; et c'est dans ce tableau que la plupart des malades sont ensuite référés dans notre institution hospitalière. Nous pouvons également évoquer la consultation tardive; le patient n’étant amené à l'hôpital qu'après des mesures de prises en charge infructueuses à domicile car, en Afrique, la majorité des personnes atteintes de diabète ne se présentent dans les centres de santé que lorsque des complications se sont déjà manifestées.

La recherche étiologique de la décompensation acidocétosique nous a justement permis d'identifier comme principal facteur déclenchant; les infections révélaient l'acidocétose chez la majorité de nos patients, ensuite vient la mauvaise observance thérapeutique. En Afrique, les infections, l'interruption thérapeutique et l'absence d’éducation thérapeutique apparaissent comme les principales causes de décompensation de l'acidocétose. Au Sénégal, Sarr [15] trouve 78% d'infections concomitantes et 69% de patients sans éducation ni suivi et 53,42% d'interruption thérapeutique. En Algérie, Boutabia [19] retrouvait une prédominance des facteurs infectieux dans 51,7% des cas. Au Bangladesh, Hossain [10] constate qu'un patient sur deux n'adhérait pas au traitement. Le taux d'interruption thérapeutique dans l’étude de Randall varie entre 56 et 78% et la plupart de ces patients n'ont donné aucune raison d'arrêt d'insulinothérapie [11].

Les sites infectieux restent les mêmes avec une répartition diversement rapportée dans la littérature. Notre étude comme celles de Sarr [15] et d'Umpierrez [20] retrouvaient une prédominance des infections urogénitales et broncho-pulmonaires.

Le taux de létalité dans notre série de 27,5% est supérieur à ceux rapportés par plusieurs auteurs [10–13, 21, 22]. Les données de la littérature suggèrent que le taux de mortalité dont est grevé l'ACD est en moyenne inférieur à 5% avec des extrêmes allant de 0 à plus de 15%, ces différences s'expliquant essentiellement par l'expérience des centres, l’âge des patients et la présence de comorbidités [1, 23]. On peut à bon droit considérer que ces données épidémiologiques sont relatives aux pays industrialisés puisqu'un peu partout en Afrique, il est rapporté un taux de mortalité plus élevé lié à l'acidocétose. Il est de 25% en Tanzanie, 33% au Kenya, 44% à Johannesburg [24]. Ce taux de mortalité élevé sur le continent africain est sans nul doute imputable à la faible couverture sanitaire du continent et aux frais de santé exorbitant [24] mais aussi aux consultations tardifs.

## Conclusion

L'acidocétose diabétique est un réel problème de santé publique à Lubumbashi avec une prévalence hospitalière de 5% et un taux de mortalité de 27,5%. Les infections, surtout celles touchant la sphère génito-urinaire ont été le principal facteur de décompensation suivi de la mauvaise observance thérapeutique. D'où la formation des diabétiques reste capitale pour la maitrise de cette affection.
